# Cultural evolution of football tactics: strategic social learning in managers’ choice of formation

**DOI:** 10.1017/ehs.2020.27

**Published:** 2020-05-21

**Authors:** Alex Mesoudi

**Affiliations:** Human Behaviour and Cultural Evolution Group, Department of Biosciences, College of Life and Environmental Sciences, University of Exeter, Penryn, Cornwall TR10 9FE, UK

**Keywords:** cultural evolution, decision-making, football, social learning, sport

## Abstract

In order to adaptively solve complex problems or make difficult decisions, people must strategically combine personal information acquired directly from experience (individual learning) and social information acquired from others (social learning). The game of football (soccer) provides extensive real world data with which to quantify this strategic information use. I analyse a 5-year dataset of all games (*n =* 9127, 2012–2017) in five top European leagues to quantify the extent to which a manager's initial formation is guided by their personal past use or success with that formation, or other managers’ use or success with that formation. I focus on the 4231 formation, the dominant formation during this period. As predicted, a manager's choice of whether to use 4231 is influenced by both their recent use of 4231 (personal information) and the use of 4231 in the entire population of managers in that division (social information). Against expectations, managers relied more on personal than social information, although this estimate was highly variable across managers and divisions. Finally, there did not appear to be an adaptive tradeoff between social and personal information use, with the relative reliance on each failing to predict managerial success.

## Graphical abstract

**Figure:**
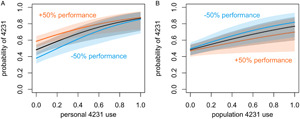

**Media summary:** When selecting formations, football managers draw on their own and others’ past use of and success with those formations

## Introduction

When solving problems or making decisions, people use a combination of personal information acquired directly from the environment (individual learning) and social information acquired by copying others (social learning) (Boyd & Richerson, 1985, 1995; Enquist, Eriksson, & Ghirlanda, 2007; Kendal et al., 2018; Laland, 2004; Perreault, Moya, & Boyd, 2012; Rogers, 1988). The strategic combination of individual and social learning is adaptive when decisions or problems are challenging, such as when environments change over time such that social information may become outdated (Boyd & Richerson, 1995; Enquist et al., 2007; Rogers, 1988), or when solutions are causally opaque or multidimensional, such that they cannot be acquired by individual learning alone and require the social learning of accumulated past solutions (Boyd & Richerson, 1985, 1995). People show this strategic mix of individual and social learning in the laboratory (Kameda & Nakanishi, 2003; McElreath et al., 2005; Mesoudi, 2008; Morgan, Rendell, Ehn, Hoppitt, & Laland, 2011; Toelch, Bruce, Newson, Richerson, & Reader, 2014; Toelch et al., 2009) and the real world (Beheim, Thigpen, & McElreath, 2014; Miu, Gulley, Laland, & Rendell, 2018) (although sometimes imperfectly: Mesoudi, 2011). When combined appropriately, individual and social learning can generate cumulative cultural evolution at the population level, where innovations generated via individual learning are preserved and accumulated over generations via social learning (Mesoudi & Thornton, 2018).

Beheim, Thigpen & McElreath (2014) provided an innovative demonstration of the strategic use of social and individual learning in the real world. They analysed decades of professional matches of the board game Go to understand the spread of an opening move, the ‘Fourfour’. This move increased rapidly in frequency from 1968 onwards. Beheim et al. showed that Go players’ use of Fourfour is predicted by both personal information, i.e. the past use and win rate of Fourfour by that player, and social information, i.e. the past use and win rate of Fourfour in the entire population of Go players. They also showed considerable between-player variation, with some players using predominantly social information (e.g. Lee Sedol) and others using mostly personal information (e.g. Takemiya Masaki, the originator of the modern Fourfour).

Here I apply the methods and approach of Beheim et al. to another competitive real world sport, football (soccer). Football is enjoyed by millions of people worldwide, and European leagues alone have a revenue of almost €30 billion (Barnard, Boor, Winn, Wood, & Wray, 2019). Football has been subject to historical analyses of tactics (Wilson, 2013b) and increasingly, by providing a wealth of fine-grained quantitative data, statistical analyses (Tamura & Masuda, 2015).

The equivalent in a football match to a Go player's opening move is a manager's starting formation. This describes how the 10 outfield players are initially organised on the pitch. Formations are typically defined by three or four numbers specifying the number of players in each segment of the pitch. For example, 442 comprises four defenders, four midfielders and two attackers. While formations may change during matches in response to player substitutions or other in-game events, all managers select one of a finite and, in practice, relatively small set of starting formations. Formations are a key component of overall tactics. For example, 541 is more defensive than 343.

The history of football tactics, crystallised in the use of different formations, is a fascinating case of cultural evolution, involving cumulative change over more than a century driven by numerous innovators from across the world, each modifying what had gone before to achieve success within the tightest of margins. The following is the briefest of narrative histories (for book length treatment, see Wilson 2013b). After the codification of the sport in Britain in the nineteenth century, football teams played in something like a 235, a very attack-heavy formation known as the ‘pyramid’. In 1925 the W-M was developed by the Arsenal manager Herbert Chapman in response to changes in the offside rule. This was 3223, which on the pitch looks like a capital W above a capital M. The Italian manager Vittorio Pozzo developed the WW (2323) in the 1930s, after which the 424 emerged seemingly independently in Brazil and Hungary in the 1950s. Alf Ramsay in England developed a 433 or 4132 formation, winning the 1966 World Cup in the process. The first ‘modern’ formation, the 442, was developed by the Russian Viktor Maslov and later used to great success by Italian managers such as Arrigo Sacchi of AC Milan in the 1980s and 1990s. Concurrently, Rinus Michels and Johan Cruyff brought considerable success to Ajax, and later Barcelona, with a modern 433. These gave way to the 4231 in the 2000s (Wilson, 2008), which in turn is being replaced (Wilson, 2013a). For example, Antonio Conte is credited with introducing a back three to the English Premier League at Chelsea in 2016–2017, to great success (Wilson, 2017).

Of course, the preceding linear narrative is highly simplified, and reality contains numerous dead-end lineages, failed experiments, ignored co-innovators and reversions to previously popular formations, just as in any evolutionary process. There have also been parallel non-formation-related innovations, from passing to pressing to improved nutrition. However, formations have remained a key part of football tactics, so much so that leading football magazines, such as *FourFourTwo* (Future Publishing, 1994 to present), are named after them. Given this, the drivers of changes in formation use is a promising subject of study for cultural evolution research.

The key question addressed here is therefore the extent to which managers use personal and social information to decide on their starting formation. This is a challenging decision, as defined above. The success of a formation depends partly on what formation the opposing manager plays, making payoffs of the same formation temporally variable and frequency dependent. Various other factors, from squad strength to luck, determine match outcomes in addition to formation, making the true contribution of the latter difficult to determine. And in the high stakes of football management (the median tenure of English Premier League managers as of August 2019 was 1 year, 158 days), there are limited opportunities to directly trial formations, especially if those trials are unsuccessful.

**Figure 1.**
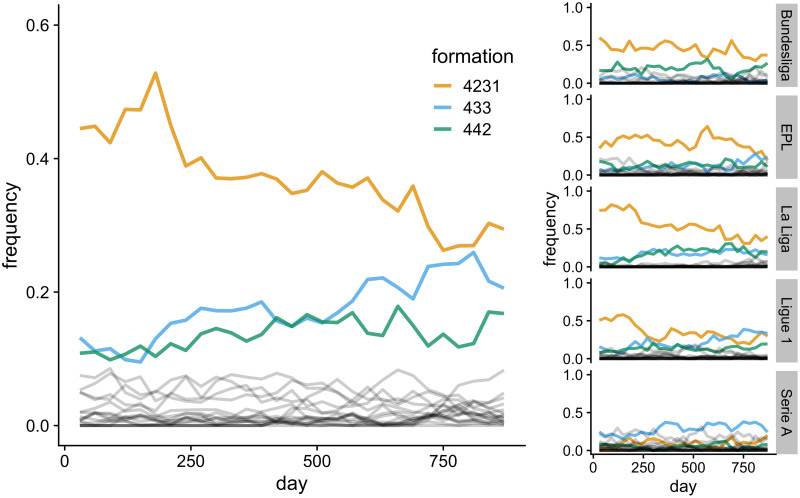
Frequencies of initial formations across all leagues (large image) and in the five separate leagues (right panels). The three most common formations are shown: 4231 (orange), 433 (blue) and 442 (green). Other less common formations are shown in grey. Frequencies are calculated as the proportion of all matches in consecutive 30-day bins that started with that formation. ‘Days’ are consecutive match days across five seasons from 2012 to 2017, omitting days on which no matches were played. EPL, English Premier League.

To maintain tractability and comparability to Beheim et al., I examine a manager's choice of whether to play the 4231 formation or not. During the period of study 4231 was the dominant formation (see Figure 1 and Wilson 2008): in the top five European domestic leagues from 2012 to 2017, it was used 37% of the time, more than double the next most common formations (18% for 433 and 14% for 442). However, 4231 also showed a clear decline in frequency during this period, from 47% in the 2012/13 season to 28% in the 2016/17 season. This decline was more extreme in some leagues than others; for example, the Spanish La Liga saw a decline in 4231 use from 78 to 37%.

Here I use a 5-year dataset of all games (*n =* 9127, 2012–2017) in the five top European leagues (English Premier League, German Bundesliga, Spanish La Liga, French Ligue 1 and Italian Serie A) to test the following hypotheses, derived from the above theory and the results of Beheim et al. (2014). All hypotheses and analyses were preregistered before running any analyses on the original data (https://osf.io/er4dx), and all data and code are available at https://github.com/amesoudi/football.

- H1: A manager's choice of whether to play 4231 is determined by a combination of personal and social information.
- H2: On average, there is greater reliance on social than personal information, as found by Beheim et al. for Go players.
- H3: There is more variation between managers in both personal and social information use compared with randomised data.
- H4: There is an *n*-shaped relationship between the ratio of population:personal information use and a manager's success, indicating that an overreliance on either form of information is less effective than strategically combining the two.

## Methods

### Data

The original dataset was downloaded from the website Kaggle, originally compiled by Jemilu Mohammed from various online sources including whoscored.com, dated 6 July 2017 (version 3) and with licence CC0: Public Domain. There are 9127 games in the dataset, which gives 18,254 starting formations (two per game, one for each team). The downloaded dataset is available as Supplementary Material.

Data was preprocessed to correct inconsistent spelling of manager names (e.g. Arsène Wenger and Arsene Wenger were merged, as were Gus Poyet and Gustavo Poyet), add one missing formation and one missing manager, add season indicators using official season start and end dates, and create predictor variables (see analysis scripts in Supplementary Material for preprocessing code).

It is important to consider the provenance and accuracy of all large secondary datasets such as this one, especially how the starting formations were coded. Opposing managers in each match officially announce their team lineups simultaneously, typically an hour before match kickoff. While they do not specify their starting formation, it is relatively straightforward to derive the formation from the announced lineup. For example, if four defenders are playing, there must be four at the back, giving a 4xxx formation. The dataset used here was compiled from whoscored.com, which in turn obtains its data from sports analysis companies such as Opta, who inform broadcasters, journalists and professional clubs in recruitment. These companies employ hundreds of analysts who are responsible for coding formations in this way. Given the importance to these companies of providing accurate data, standardised definitions of formations are used which hopefully means that the data used here reliably represents the actual formations used. Nevertheless, bias or error can never be completely avoided in large datasets that ultimately involve some human interpretation, so replication with alternative datasets is encouraged.

### Predictors

Following Beheim et al., predictors were created using a moving time window of *X* days previous to the formation choice in question. That is, for each formation choice, predictors were calculated from all games played in the previous *X* days, not including the day on which that formation was played. In the analyses reported below *X* = 30, and analyses are repeated in the Supplementary Material using *X* = 20, *X* = 40 and *X* = 60 (these choices were preregistered, and generated qualitatively identical results to those presented below for *X* = 30). The *X*-day window was reset at the start of each season, so games played in the first *X* days of each season were not included in the analyses. The Bundesliga, unlike the other leagues, has a mid-season break of more than 30 days; this was reduced to 10 days in each season so that all *X*-day windows yielded prior game data.

Personal predictors were: (a) personal use of 4231 – the proportion of games in the *X*-day window played by that manager in that division and season in which 4231 was chosen, out of all games played by that manager in that division and season in the *X*-day window, centred on 0.5; (b) personal 4231 win rate – the proportion of games played with 4231 by that manager in that division and season in the *X*-day window that were won, centred on the equivalent win rate for games played with a non-4231 formation; (c) the interaction between personal 4231 use and personal 4231 win rate; and (d) the interaction between personal 4231 use and the managers’ overall win rate with any formation, with the latter centred on the overall win rate of all managers in that division and season.

Population predictors were: (e) population 4231 use – the proportion of games in the *X*-day window played in that division and season in which 4231 was chosen, out of all games played in that division and season in the *X*-day window, centred on 0.5; (f) population 4231 win rate – the proportion of games played with 4231 in that division and season in the *X*-day window that were won, centred on the equivalent win rate for games played with a non-4231 formation; (g) the interaction between population 4231 use and population 4231 win rate; and (h) the interaction between population 4231 use and the managers’ overall win rate with any formation, with the latter centred on the overall win rate of all managers in that division and season.

Additional predictors were an indicator variable denoting whether the formation was used home or away, and a measure of team strength which was the proportion of games won by that team in that entire season, centred on the mean win rate of all teams in that division in that season.

### Analyses

Bayesian multi-level regression models were run using the rethinking package (McElreath, 2016, 2019). All models contained varying effects for manager and division. The null model contained only the home/away and team strength predictors. The personal model contained home/away, team strength and the four personal information predictors. The population model contained home/away, team strength and the four population information predictors. The full model contained home/away, team strength and all eight personal and population variables. In addition to varying intercepts for manager and division, the personal model contained varying slopes for personal information use and win rate, the population model contained varying slopes for population information use and win rate and the full model had both sets of varying slopes. Full model specifications can be found in the Supplementary Material.

### Predictions

Hypotheses H1–H4 specified in the Introduction were tested statistically via the following predictions.

#### H1 predictions

The full regression model with personal (individual) and population (social) predictors has better fit to the data than the personal-only model, the population-only model and the null model with neither personal nor population predictors. Fit is indicated by model comparison using WAIC. Additionally, in the full model, there are effects of (a) personal 4231 use, (b) personal 4231 win rate, (c) population 4231 use and (d) population 4231 win rate, and interactions between (e) personal 4231 use and win, and between (f) population 4231 use and win rates. Effects are indicated by the parameter estimates’ 89% confidence interval (CI; specifically, 89% percentile interval, see McElreath, 2016) not including zero in the full model.

#### H2 prediction

The ratio of population:personal use, calculated by dividing the estimate for population use in the full model by the estimate for personal use in the full model, is reliably >1, as indicated by 89% CIs not overlapping 1.

#### H3 predictions

The standard deviation of the varying effects for managers’ (a) personal 4231 use and (b) population 4231 use in the best-supported regression model will be larger than the equivalent standard deviations in a model generated with dummy data that has randomised formation and win rates across managers.

#### H4 prediction

In a regression model with manager as unit of analysis which contains the managers’ population:personal information use ratio and their personal win rate relative to other managers in that division and season, the win rate is reliably predicted by the square of the population:personal use ratio (i.e. a negative coefficient in a quadratic polynomial).

## Results

### H1 predictions: combination of population and personal information use

As predicted, the full model containing both personal and population predictors was best supported, containing all of the model weight compared with the personal, population and null models (Table 1). However, the WAIC of the personal model came much closer to the full model WAIC than those of the population or null models.

**Table 1.** Model comparison to test hypothesis H1. WAIC = Widely Applicable Information Criterion; pWAIC = penalty term for WAIC; dWAIC =difference from WAIC of best model; SE = standard error; dSE = difference from SE of best model

	WAIC	pWAIC	dWAIC	Weight	SE	dSE
Full model	12273.11	338.30	0.00	1	144.03	NA
Personal model	12346.35	298.20	73.24	0	144.19	15.16
Population model	13547.72	356.86	1274.61	0	137.23	88.05
Null model	14331.91	251.01	2058.79	0	135.11	101.69

Table 2 shows the parameter estimates for the full model. As predicted, there are effects of personal 4231 use, personal 4231 win rate and an interaction between these two predictors. There is also an effect of population 4231 use. However, there were no reliable effects of population 4231 win rate, nor interactions between personal 4231 use and personal overall win rate, nor interactions between population 4231 use and either population 4231 win rate or personal overall win rate.

**Table 2.** Parameter estimates for the full model. Home/away is an indicator trait with separate estimates for formations used home and away. Varying effects show the standard deviations of the varying intercepts and slopes. See the Supplementary Material for full model specification and priors

	Mean	SD	5.5% CI	94.5% CI
*Fixed effects*				
Home/away: home	−0.01	0.17	−0.27	0.24
Home/away: away	−0.14	0.17	−0.41	0.11
Team strength	0.03	0.27	−0.39	0.46
Personal 4231 use	2.08	0.63	0.96	2.92
Personal 4231 win rate	0.84	0.13	0.63	1.06
Personal 4231 use × personal 4231 win rate	−0.64	0.21	−0.98	−0.30
Personal 4231 use × personal win rate	0.11	0.35	−0.46	0.66
Population 4231 use	1.34	0.48	0.55	2.03
Population 4231 win rate	−0.11	0.20	−0.43	0.22
Population 4231 use × population 4231 win rate	−0.62	0.67	−1.69	0.45
Population 4231 use × personal win rate	−1.07	0.51	−1.87	−0.24
*Varying effects*				
Manager	0.71	0.07	0.60	0.83
Manager × personal 4231 use	1.26	0.14	1.04	1.50
Manager × population 4231 win rate	2.00	0.42	1.35	2.67
Division	0.27	0.19	0.05	0.62
Division × personal 4231 use	1.25	0.59	0.57	2.32
Division × population 4231 win rate	0.69	0.53	0.06	1.66

**Figure 2.**
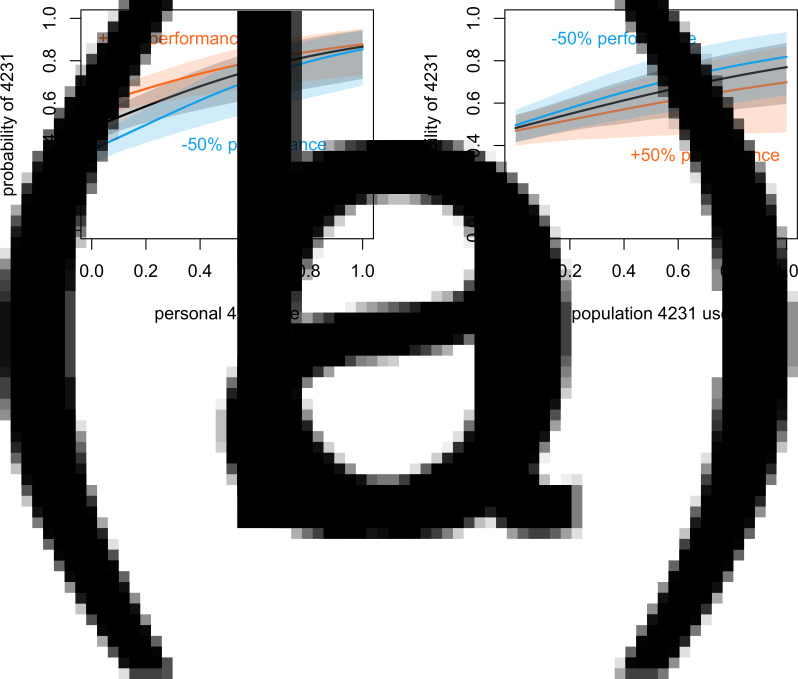
(a) The predicted probability of using 4231 as a function of personal 4231 use, assuming that the 4231 win rate is the same as the non-4231 rate (black line, grey shading showing 89% CI), assuming that the personal 4231 win rate is 50% higher than the non-4231 win rate (orange line and shading), and assuming that the personal 4231 win rate is 50% lower than the non-4231 win rate (blue line and shading). (b) The equivalent predictions for population 4231 use, and + 50% or −50% population 4231 win rates relative to non-4231 population win rates.

Figure 2 shows how past personal 4231 use (Figure 2a) and population 4231 use (Figure 2b) increase the probability of 4231 being chosen. The interaction between personal 4231 use and personal 4231 win rate revealed in Table 2 can be seen in Figure 2a: for managers who have seldom used 4231 in recent games, a higher win rate with 4231 increases their likelihood of choosing 4231, and a lower win rate decreases that likelihood. Also consistent with Table 2, Figure 2b reveals no reliable interaction between population 4231 use and population 4231 win rate, with the higher and lower performance lines falling within the average performance CI shading.

### H2 predictions: ratio of population to personal 4231 use

The ratio of population:personal use, as calculated using the full model (see Table 2), had a mean of 0.68 (89% CI [0.24, 1.41]). Contrary to hypothesis H2, this suggests that personal information was more influential than population (i.e. social) information. As indicated by the wide confidence intervals, however, this estimate was highly uncertain, and there was a lot of variation in this ratio across managers and divisions.

### H3 predictions: variation across managers and divisions

Table 3 shows that, as predicted, there was more variation across managers in the effects of both personal 4231 use and population 4231 use compared with randomised data. Also as predicted, there was more variation across divisions in the effect of personal 4231 use compared with randomised data, but not in the effect of population 4231 use.

**Table 3.** Tests of the differences between varying effects from the real data and varying effects from randomised data, to test hypothesis H3. Values shown are real minus randomised standard deviations

	Mean	SD	5.5% CI	94.5% CI
Manager × personal 4231 use	1.07	0.19	0.78	1.37
Manager × population 4231 use	1.78	0.46	1.04	2.48
Division × personal 4231 use	1.16	0.59	0.45	2.24
Division × population 4231 use	0.46	0.58	-0.27	1.47

### H4 predictions: population to personal use ratio and win rate

**Figure 3.**
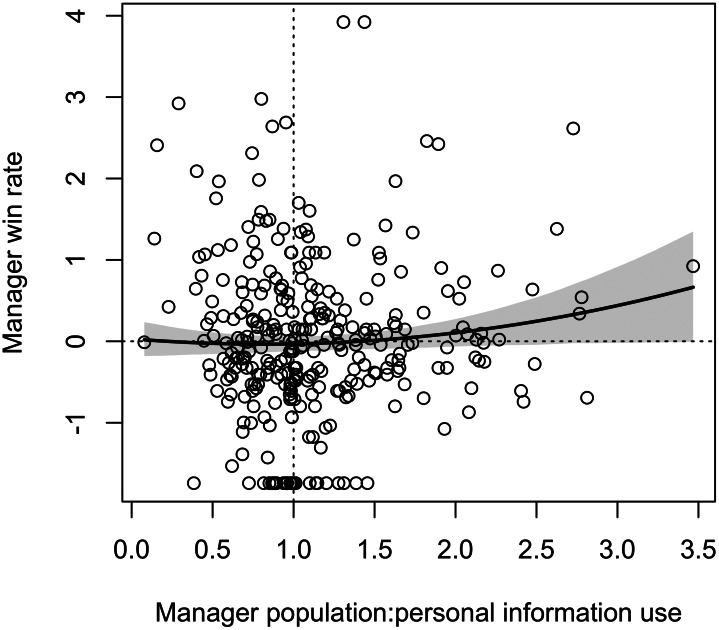
Relationship between each manager's win rate relative to the average manager's win rate and each manager's population:personal information use ratio as generated from the full model. Dotted lines indicate the average win rate and equal ratio. The thick line shows the predicted mean win rate at each value of the ratio, with shaded 89% CIs.

Contrary to hypothesis H4, there was no *n*-shaped quadratic relationship between manager win rate and population:personal information use ratio (Table 4 and Figure 3). There was also no reliable positive or negative linear relationship: as shown in Figure 3, for most use ratios the relationship with win rate is flat. Managers with very high ratios, indicating more reliance on population 4231 use than personal 4231 use, had higher win rates, but the shaded 89% CIs always included zero, and this increase is probably unduly influenced by the right-most outlier.

**Table 4.** Model estimates for the quadratic regression model with manager as unit of analysis, to test hypothesis H4. Parameter *a* is the intercept, *b*_1_ is the linear coefficient and *b*_2_ the quadratic coefficient. Win rate is modelled as normally distributed with standard deviation sigma. See the Supplementary Material for priors

	Mean	SD	5.5% CI	94.5% CI
*a*	0.03	0.14	−0.19	0.26
*b*_1_	−0.16	0.18	−0.44	0.11
*b*_2_	0.10	0.07	−0.01	0.21
Sigma	1.00	0.04	0.94	1.06

### Exploratory analysis: between division effects

**Figure 4.**
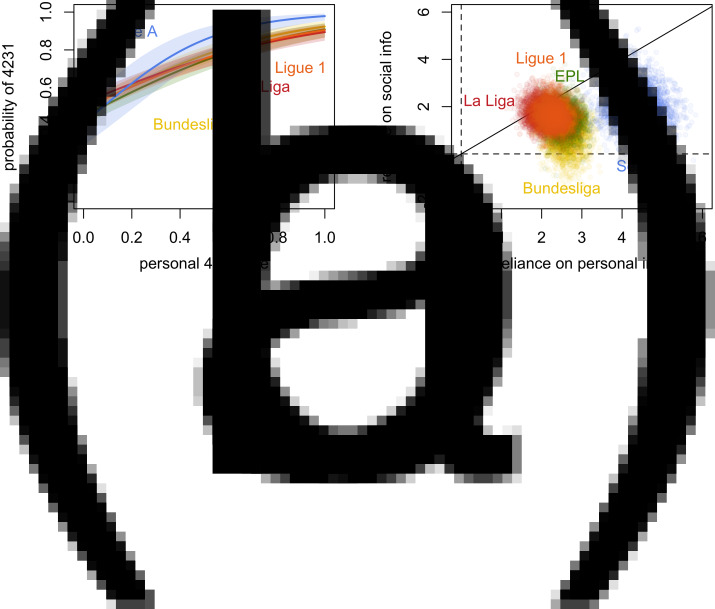
(a) Effect of personal 4231 use on probability of choosing 4231 broken down by division. (b) Joint posterior densities of the relative reliance on personal and social information, for the five divisions. The solid black diagonal indicates equal personal and social influence. EPL, English Premier League.

Figure 4 shows the variation across the five divisions in the effect of personal 4231 use. Four of the divisions are almost identical. The Italian Serie A, however, shows a much stronger effect of personal information use. This is likely because of the low overall frequency of 4231 in this division, as shown in the Serie A inset in Figure 1. The majority of managers in Serie A never or seldom used 4231 during this period. Out of 67 managers who managed in Serie A, only three used 4231 in more than 50% of their games, only 10 used it in more than 25% of their games, and 35 never used it. The small number of managers who used 4231 in a majority of their games would have disproportionately influenced the model's predicted probability of subsequently picking 4231 at high values of personal 4231 use, as seen in Figure 4a.

### Exploratory analysis: between manager effects

**Figure 5.**
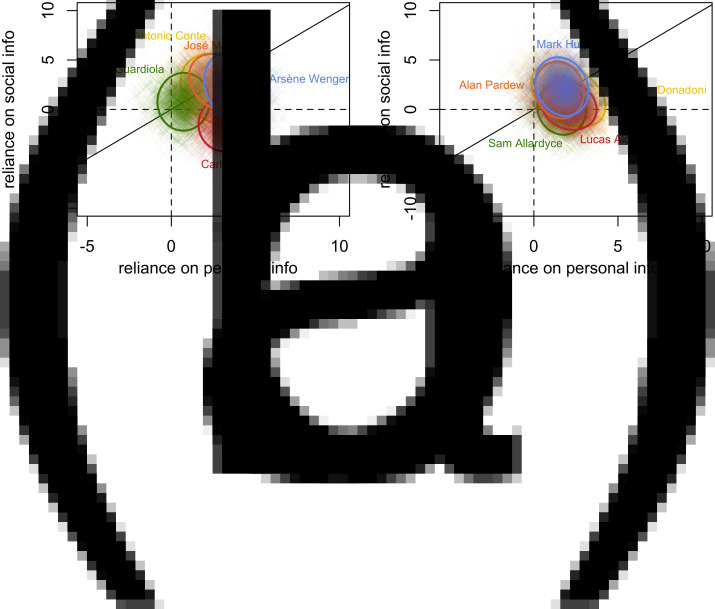
Joint posterior densities of the relative reliance on personal and social information, for (a) five managers with high win rates and (b) five managers with low win rates, all of whom have managed more than 100 games in the period of study. Ellipses indicate the 80% confidence region for each manager. The solid black diagonals indicate equal personal and social influence.

Figure 5a shows variation across five successful managers who played over 100 games in our study period: Antonio Conte (78% win rate, 101 games), Josep Guardiola (74% win rate, 124 games), Carlo Ancelotti (73%, 131 games), José Mourinho (59% win rate, 149 games) and Arsene Wenger (59% win rate, 170 games).

Figure 5b shows variation across five relatively unsuccessful managers who played over 100 games in our study period: Roberto Donadoni (30% win rate, 161 games), Sam Allardyce (32% win rate, 151 games), Alan Pardew (34%, 147 games), Lucas Alcaraz (27% win rate, 107 games) and Mark Hughes (35% win rate, 144 games).

While exploratory, we can see in these figures that successful managers seem to be more different from one another in information use strategies than unsuccessful managers. Carlo Ancelotti has less reliance on social information than the other successful managers, while Josep Guardiola has relatively less reliance on personal information. The unsuccessful managers show substantial overlap with one another over a smaller combined area than the successful managers. Whether this pattern is robust, and the reasons for it, are worthy of further study. Perhaps there are more ways to be successful than there are to be unsuccessful in football management.

### Additional analyses

The reviewers and editor raised two concerns about the preregistered models described above. These were addressed by re-running the models with slightly different specifications. Results for these re-analyses are presented in the Supplementary Material. Neither re-analysis yielded results that were qualitatively different from those found using the original preregistered analyses presented above, supporting the robustness of these findings.

The first concern was that the population predictors (use and win rate of 4231 across the entire league in the *X*-day period) contained formations used by that same manager for that same team. These formations would have entered into both the personal and population predictors, such that the social information would have also included personal information. The models were therefore re-run excluding formations used by the same team during the *X*-day window. That is, for team *i*, the population predictors are calculated from all formations used in the *X*-day window except those used by team *i*. This change had negligible effects on the results, and all conclusions for all hypotheses remain qualitatively identical to the original findings derived from the original preregistered analyses presented above (see Supplementary Material).

The second concern was the lack of controls related to the opponent team in a match. First, it seems reasonable to assume that managers might change their formation based on the strength of the opponent, playing more defensive formations against strong teams and attacking formations against weak teams. Second, managers might change their formation in response to the anticipated formation played by the opponent. Because team lineups are announced simultaneously, managers cannot know for sure what formation the opposing manager will play. However, they can perhaps guess based on past formations. Specifically related to 4231, managers may attempt to counter 4231 with either the same formation, matching players in the same positions, or with a different formation, in an attempt to break the 4231 domination. To address both these points, the models were re-run including (a) the relative strength of the opponent team, calculated in the same way as the own team strength predictor and (b) the formation played by the opponent, coded as 4231 or non-4231. Including these controls had negligible effect on the parameter estimates, and did not qualitatively change conclusions regarding any of the hypotheses compared with the original preregistered analyses presented above (see Supplementary Material).

## Discussion

Complex decisions often require the strategic combination of personal information acquired via individual learning and population-wide information acquired via social learning, each of which has distinct advantages and disadvantages. Beheim et al. (2014) analysed decades of games of Go to show that professional Go players combine personal and social information when deciding on opening moves, and these individual-level strategic decisions generated long-term evolutionary dynamics. Here, I applied the same methodological approach to the game of football, where the equivalent to an opening Go move is a manager's choice of starting formation. Consequently, I examined personal and social influences on a manager's choice of whether to use the most popular 4231 formation or not.

Over five seasons from 2012 to 2017 across the five top European leagues, it is indeed the case (supporting Hypothesis H1) that a manager's choice of whether to play 4231 is on average predicted by both their own recent use of 4231 (personal information) and the frequency with which 4231 is recently used in the entire population of managers from the same league (social information), as well as the manager's personal win rate with 4231.

Contrary to the more specific prediction (Hypothesis H2) that managers should rely more on social than personal information, given the difficulty of personally trialling different formations in the high stakes world of football management and previous findings of greater social information use by Beheim et al. (2014), there was if anything more reliance on personal information. This is puzzling not only for the aforementioned reasons (the difficulty of individual learning should favour reliance on social learning, plus the previous findings of Beheim et al.), but also the fact that the population provides much more information overall in the same time period. For the 30-day time window used here, a manager using population-wide information can draw on a mean of 76 games played across the entire division, while personal information only provides data from a mean of 3.6 games. The preference for personal information may be evidence for an egocentric bias, with managers weighting their own experience higher than others’. Previous laboratory experiments have found similar over-reliance on individual learning at the expense of social learning (Efferson, Lalive, Richerson, McElreath, & Lubell, 2008; McElreath et al., 2005; Mesoudi, 2011; Morgan et al., 2011; Toelch et al., 2014; Weizsacker, 2010).

On the other hand, such estimates of the relative reliance on personal and population information were very uncertain, with confidence intervals so wide as to be consistent with a reliance on either form. This is due to the extensive variation in information use strategy across both managers and divisions, much more than would be expected if decisions were random (Hypothesis H3).

However, this variation does not seem to exhibit an adaptive tradeoff (Hypothesis H4). Managers with different ratios of population:personal information use did not vary in their success, contrary to the expectation that an overreliance on either type of information should be detrimental. Perhaps in team sports like football, starting formations do not reliably translate into success in the way that opening moves in Go do, given the many other factors that determine success and the possibility of changing formations during a game.

Exploratory analyses showed that one league, Serie A, showed a much stronger effect of personal information than the other leagues. This is probably because of the low overall use of 4231 in this league, with very few managers using this formation; this small number of managers drove the effect, given that if a manager used 4231 previously, they were highly likely to be one of the few managers to use it in the future. This illustrates two points: first, the importance of including league (or any other relevant grouping variable) as a varying effect in the analysis, to account for unusual patterns such as this; and second, the influence of overall trait frequency on the learning strategies that are employed. Rare traits may be influenced more by personal experience, when a manager is unable to draw on the experience of others.

In this study I have, following Beheim et al. (2014), framed my hypotheses and findings in terms of social/individual learning. That is, I assume that if past use or past success predicts a manager's formation choice, this is indicative of that manager learning, either individually or socially, that that formation is effective. However, it is always challenging to use observational data to draw causal inferences regarding social interactions or peer effects (Angrist, 2014; Manski, 2000). Alternative explanations should always be considered, and are difficult to rule out. For example, it is possible that exogenous events such as rule changes could generate concerted change in managers’ formation choices. While this might look like it is caused by the social learning of formation use, adoption might be entirely independent of other managers. As noted in the Introduction, a change in the offside rule in the 1920s did indeed lead to the appearance and spread of a new, more defensive formation (although in that case, informal accounts suggest that there was in fact social learning from a single innovator, Herbert Chapman). However, the lack of any significant rule changes during the period of study of the present analysis makes it unlikely that the current findings can be easily explained in terms of exogenous rule changes.

A more plausible alternative is that formation choice is subject to coordination incentives, in a way that perhaps Go opening moves are not. While only one Go player makes an opening move in a game, in a football match both managers select a starting formation. It is therefore possible that a formation might be chosen in response to the choice of the opposing manager. Consequently, all managers might begin the season with contingent strategies (e.g. play 4231 against 4231, otherwise play 433 against weak opponents and 451 against strong opponents), and changes observed during the season are simply different contingent rules being implemented in response to accumulating information about opposing managers’ likelihood of using a particular formation, rather than the learning of new formations or formation effectiveness. While this may indeed be an added consideration in football compared with Go, it is unlikely to account for all of the findings presented above. Typically, lineups and the likely formations are announced simultaneously by both managers prior to the game. This means that the initial formation choice cannot be a direct response to the other manager's formation, only to what the manager anticipates the other manager will play. Furthermore, as shown in the Supplementary Material, additional models containing both opponent formation and opponent strength did not qualitatively change the results. Nevertheless, future analyses might more explicitly incorporate these coordination incentives.

One reason for the absence of an adaptive tradeoff between personal and social information use, as well as the lower than expected reliance on social information, might be that football is a team sport and, unlike individual pursuits such as Go, subject to collective action problems. One might expect managers to use formations that fit the players available to them. Managers with access to a Lionel Messi will build their team around such star players. Managers whose strikers are all injured will be forced to use a more defensive formation than they otherwise would. If managers have different players available, then copying the formation of other managers will be less viable compared with a Go player, who can easily copy an opening move from another player. Framed in this way, it is all the more surprising that there was any signal of social information use at all in the current study. Yet this is consistent with observations of the ‘natural history’ of football formation use. As described in the Introduction, specific managers are frequently identified with specific formations. Antonio Conte brought a back-three to English football with Chelsea, fitting the Chelsea players into such a system rather than adapting his formation to the Chelsea players he had available. Given his success, other managers copied this innovation. Nevertheless, further exploration of how social information use differs across individual and collective sports would be valuable. Team sports in which managerial influence is weaker than it is in football might not find any social information use signal at all.

Future analyses might apply more generative models to data such as this, for example reinforcement, memory decay or Bayesian updating models, to more directly model how managers might be updating their beliefs in response to constantly changing personal and social information (McElreath et al., 2005; Perreault et al., 2012). This may necessitate different implementations of the time window. Here this was a fixed *X*-day time window preceding the formation choice. More advanced models might include all previous games in a season/division weighted by recency. Models might also incorporate social network ties amongst managers to test the extent to which influence flows along network ties. For example, several managers in the dataset have cited Marcelo Bielsa as a major influence on their tactics (e.g. Mauricio Pochettino, Diego Simeone, Mauricio Pellegrino, Pep Guardiola), many of whom are Bielsa's former players.

In conclusion, this study represents one of several recent attempts to apply theories from the field of cultural evolution to large, real-world datasets that comprise individual-level choices, preferences and decisions that may be influenced by both social and individual learning (Beheim et al., 2014; Brand, Acerbi, & Mesoudi, 2019; Miu et al., 2018; Youngblood, 2019). A closer interplay between real-world data, model-driven theoretical considerations and laboratory experiments can provide a broader understanding of human cultural adaptation, applicable not just to sports and boardgames but also to any pursuit where individual and social information can be combined to inform complex decisions.

## Data Availability

All data and analysis scripts are openly available at https://github.com/amesoudi/football.

## Supplementary material

For supplementary material accompanying this paper visit http://dx.doi.org/10.1017/ehs.2020.27. click here to view supplementary material
