# Supplementary material for: Cultural evolution of football tactics: strategic social learning in managers’ choice of formation
Source: Evol Hum Sci. 2020 May 21;2:e25. doi: 10.1017/ehs.2020.27 (PMC10427434; doi:10.1017/ehs.2020.27)
Supplement: Supplementary file 1 [file S2513843X20000274sup001.pdf]

Supplementary Material for ‘Cultural evolution of football tactics: Strategic social learning in managers’ choice of formation’

Alex Mesoudi *

30 March, 2020

Contents

1 Model specifications **3**

1.1 Null model 3

1.2 Personal model 3

1.3 Population model 4

1.4 Full model 5

1.5 Manager model 6

2 Results with different predictor time windows **7**

2.1 Hypothesis H1: combination of population and personal information use 7

2.2 Hypothesis H2: ratio of population to personal 4231 use 11

2.3 Hypothesis H3: variation across managers and divisions 11

2.4 Hypothesis H4: population to personal use ratio and win rate 12

*Human Behaviour and Cultural Evolution Group, Department of Biosciences, College of Life and Environmental Sciences, University of Exeter, Penryn, Cornwall TR10 9FE, United Kingdom; a.mesoudi@exeter.ac.uk

3 Revised models excluding own team formations from population predictors **13**

3.1 Hypothesis H1: combination of population and personal information use 13

3.2 Hypothesis H2: ratio of population to personal 4231 use 13

3.3 Hypothesis H3: variation across managers and divisions 15

3.4 Hypothesis H4: population to personal use ratio and win rate 15

4 Revised models including additional opponent controls **16**

4.1 Hypothesis H1: combination of population and personal information use 16

4.2 Hypothesis H2: ratio of population to personal 4231 use 18

4.3 Hypothesis H3: variation across managers and divisions 18

4.4 Hypothesis H4: population to personal use ratio and win rate 18

References **19**

1 Model specifications

The following code gives the models as specified in the rethinking package[1] in R, using non-centred parameterisation.

1.1 Null model

```
fourtwothreeone ~ dbinom(1, p)
logit(p) <- a_homeaway[homeaway] + b_team_strength * team_strength +
    a_manager[manager_id] * sigma_manager + a_division[division_id] *
    sigma_division
a_homeaway[homeaway] ~ dnorm(0, 1)
b_team_strength ~ dnorm(0, 1)
a_manager[manager_id] ~ dnorm(0, 1)
a_division[division_id] ~ dnorm(0, 1)
sigma_manager ~ dexp(1)
sigma_division ~ dexp(1)
```

1.2 Personal model

```
fourtwothreeone ~ dbinom(1, p)
logit(p) <- a_homeaway[homeaway] + b_team_strength * team_strength +
    a_manager[manager_id, 1] + a_manager[manager_id, 2] * pers_use +
    a_division[division_id, 1] + a_division[division_id, 2] *
    pers_use + b_pers_use * pers_use + b_pers_win * pers_win +
    b_pers_useXpers_win * pers_useXpers_win + b_pers_useXpers_win_all *
    pers_useXpers_win_all
a_homeaway[homeaway] ~ dnorm(0, 1)
b_team_strength ~ dnorm(0, 1)
```

```
transpars > matrix[manager_id, 2]:a_manager <- compose_noncentered(sigma_manager,
    L_Rho_manager, z_manager)
transpars > matrix[division_id, 2]:a_division <- compose_noncentered(sigma_division,
    L_Rho_division, z_division)
matrix[2, manager_id]:z_manager ~ normal(0, 1)
matrix[2, division_id]:z_division ~ normal(0, 1)
b_pers_use ~ dnorm(0, 1)
b_pers_win ~ dnorm(0, 1)
b_pers_useXpers_win ~ dnorm(0, 1)
b_pers_useXpers_win_all ~ dnorm(0, 1)
vector[2]:sigma_manager ~ dexp(1)
cholesky_factor_corr[2]:L_Rho_manager ~ lkj_corr_cholesky(2)
vector[2]:sigma_division ~ dexp(1)
cholesky_factor_corr[2]:L_Rho_division ~ lkj_corr_cholesky(2)
```

1.3 Population model

```
fourtwothreeone ~ dbinom(1, p)
logit(p) <- a_homeaway[homeaway] + b_team_strength * team_strength +
    a_manager[manager_id, 1] + a_manager[manager_id, 2] * pop_use +
    a_division[division_id, 1] + a_division[division_id, 2] *
    pop_use + b_pop_use * pop_use + b_pop_win * pop_win + b_pop_useXpop_win *
    pop_useXpop_win + b_pop_useXpers_win_all * pop_useXpers_win_all
a_homeaway[homeaway] ~ dnorm(0, 1)
b_team_strength ~ dnorm(0, 1)
transpars > matrix[manager_id, 2]:a_manager <- compose_noncentered(sigma_manager,
    L_Rho_manager, z_manager)
transpars > matrix[division_id, 2]:a_division <- compose_noncentered(sigma_division,
    L_Rho_division, z_division)
```

```
matrix[2, manager_id]:z_manager ~ normal(0, 1)
matrix[2, division_id]:z_division ~ normal(0, 1)
b_pop_use ~ dnorm(0, 1)
b_pop_win ~ dnorm(0, 1)
b_pop_useXpop_win ~ dnorm(0, 1)
b_pop_useXpers_win_all ~ dnorm(0, 1)
vector[2]:sigma_manager ~ dexp(1)
cholesky_factor_corr[2]:L_Rho_manager ~ lkj_corr_cholesky(2)
vector[2]:sigma_division ~ dexp(1)
cholesky_factor_corr[2]:L_Rho_division ~ lkj_corr_cholesky(2)
```

1.4 Full model

```
fourtwothreeone ~ dbinom(1, p)
logit(p) <- a_homeaway[homeaway] + b_team_strength * team_strength +
    a_manager[manager_id, 1] + a_manager[manager_id, 2] * pers_use +
    a_manager[manager_id, 3] * pop_use + a_division[division_id,
    1] + a_division[division_id, 2] * pers_use + a_division[division_id,
    3] * pop_use + b_vars
b_vars <- b_pers_use * pers_use + b_pers_win * pers_win + b_pers_useXpers_win *
    pers_useXpers_win + b_pers_useXpers_win_all * pers_useXpers_win_all +
    b_pop_use * pop_use + b_pop_win * pop_win + b_pop_useXpop_win *
    pop_useXpop_win + b_pop_useXpers_win_all * pop_useXpers_win_all
a_homeaway[homeaway] ~ dnorm(0, 1)
b_team_strength ~ dnorm(0, 1)
transpars > matrix[manager_id, 3]:a_manager <- compose_noncentered(sigma_manager,
    L_Rho_manager, z_manager)
transpars > matrix[division_id, 3]:a_division <- compose_noncentered(sigma_division,
    L_Rho_division, z_division)
```

```
matrix[3, manager_id]:z_manager ~ normal(0, 1)
matrix[3, division_id]:z_division ~ normal(0, 1)
b_pers_use ~ dnorm(0, 1)
b_pers_win ~ dnorm(0, 1)
b_pers_useXpers_win ~ dnorm(0, 1)
b_pers_useXpers_win_all ~ dnorm(0, 1)
b_pop_use ~ dnorm(0, 1)
b_pop_win ~ dnorm(0, 1)
b_pop_useXpop_win ~ dnorm(0, 1)
b_pop_useXpers_win_all ~ dnorm(0, 1)
vector[3]:sigma_manager ~ dexp(1)
cholesky_factor_corr[3]:L_Rho_manager ~ lkj_corr_cholesky(2)
vector[3]:sigma_division ~ dexp(1)
cholesky_factor_corr[3]:L_Rho_division ~ lkj_corr_cholesky(2)
```

1.5 Manager model

```
win_rate ~ dnorm(mu, sigma)
mu <- a + b1 * info_ratio + b2 * info_ratio_sq
a ~ normal(0, 1.5)
b1 ~ normal(0, 0.2)
b2 ~ normal(-0.1, 0.2)
sigma ~ dexp(1)
```

Table S1: Model comparison to test hypothesis H1, with X=20 day time window.

	WAIC	pWAIC	dWAIC	weight	SE	dSE
Full model	12721.79	391.68	0.00	1	146.28	NA
Personal model	12850.05	335.69	128.26	0	146.38	22.23
Population model	14043.59	360.30	1321.80	0	138.68	85.10
Null model	14763.00	251.70	2041.21	0	136.94	99.36

Table S2: Model comparison to test hypothesis H1, with X=40 day time window.

	WAIC	pWAIC	dWAIC	weight	SE	dSE
Full model	11723.16	321.92	0.00	1	139.83	NA
Personal model	11811.48	278.71	88.32	0	139.95	15.87
Population model	12835.30	353.70	1112.14	0	134.26	84.39
Null model	13642.63	248.61	1919.47	0	131.65	98.84

2 Results with different predictor time windows

The analyses in the main text use a time window of $X = 30$ days to calculate all use and win rate predictors. Here I present results with different time windows: $X = 20$, $X = 40$ and $X = 60$ (NB these choices, both what to include in the main paper and alternatives to consider here, were all preregistered).

2.1 Hypothesis H1: combination of population and personal information use

Tables S1-S3 show model comparisons for X=20, X=40 and X=60 day windows respectively, to compare with Table 1 in the main paper that uses X=30. Across all time windows, the full model is best supported, receiving all model weight, and the personal model was next best supported.

Tables S4-S6 show the full models for X=20, X=40 and X=60 day windows, to compare with Table 2 in the main paper that uses X=30. Apart from inevitable differences in coefficient values, the models all have the same patterns of results: for each time window, the same predictors either cross zero or do not cross zero.

Table S3: Model comparison to test hypothesis H1, with X=60 day time window.

	WAIC	pWAIC	dWAIC	weight	SE	dSE
Full model	10888.90	301.13	0.00	1	133.29	NA
Personal model	10995.13	250.25	106.23	0	133.51	16.74
Population model	11731.49	344.18	842.59	0	128.96	76.88
Null model	12570.19	241.00	1681.29	0	126.57	92.94

Table S4: Parameter estimates for the full model, with X=20 day time window. Home/away is an indicator trait with separate estimates for formations used home and away. Varying effects show the standard deviations of the varying intercepts and slopes. See SI for full model specification and priors.

	mean	sd	5.5%	94.5%
Fixed effects:				
Home/away: Home	-0.15	0.25	-0.54	0.26
Home/away: Away	-0.26	0.25	-0.65	0.14
Team strength	0.15	0.28	-0.30	0.58
Personal 4231 use	1.82	0.49	0.98	2.48
Personal 4231 win rate	0.50	0.14	0.28	0.72
Personal 4231 use * personal 4231 win rate	-0.31	0.15	-0.54	-0.08
Personal 4231 use * personal win rate	0.05	0.30	-0.42	0.54
Population 4231 use	1.52	0.44	0.80	2.16
Population 4231 win rate	-0.17	0.15	-0.41	0.08
Population 4231 use * population 4231 win rate	-0.60	0.54	-1.47	0.26
Population 4231 use * personal win rate	-0.42	0.37	-1.00	0.15
Varying effects:				
Manager	1.02	0.08	0.89	1.15
Manager * personal 4231 use	1.05	0.12	0.88	1.25
Manager * population 4231 win rate	2.62	0.34	2.08	3.17
Division	0.53	0.27	0.22	1.00
Division * personal 4231 use	0.95	0.45	0.46	1.80
Division * population 4231 win rate	0.56	0.51	0.04	1.48

Table S5: Parameter estimates for the full model, with X=40 day time window. Home/away is an indicator trait with separate estimates for formations used home and away. Varying effects show the standard deviations of the varying intercepts and slopes. See SI for full model specification and priors.

	mean	sd	5.5%	94.5%
Fixed effects:				
Home/away: Home	0.04	0.14	-0.18	0.26
Home/away: Away	-0.09	0.14	-0.31	0.12
Team strength	0.08	0.27	-0.35	0.52
Personal 4231 use	2.16	0.68	0.92	3.06
Personal 4231 win rate	0.92	0.15	0.67	1.17
Personal 4231 use * personal 4231 win rate	-0.97	0.25	-1.37	-0.57
Personal 4231 use * personal win rate	0.29	0.41	-0.38	0.95
Population 4231 use	1.33	0.53	0.40	2.07
Population 4231 win rate	0.01	0.23	-0.35	0.38
Population 4231 use * population 4231 win rate	0.01	0.73	-1.14	1.17
Population 4231 use * personal win rate	-1.58	0.57	-2.50	-0.67
Varying effects:				
Manager	0.66	0.07	0.54	0.78
Manager * personal 4231 use	1.32	0.15	1.08	1.55
Manager * population 4231 win rate	2.22	0.44	1.54	2.91
Division	0.22	0.18	0.03	0.53
Division * personal 4231 use	1.31	0.63	0.57	2.48
Division * population 4231 win rate	0.73	0.56	0.06	1.76

Table S6: Parameter estimates for the full model, with X=60 day time window. Home/away is an indicator trait with separate estimates for formations used home and away. Varying effects show the standard deviations of the varying intercepts and slopes. See SI for full model specification and priors.

	mean	sd	5.5%	94.5%
Fixed effects:				
Home/away: Home	0.05	0.13	-0.15	0.23
Home/away: Away	-0.11	0.13	-0.30	0.08
Team strength	0.17	0.28	-0.28	0.62
Personal 4231 use	2.20	0.62	1.05	3.01
Personal 4231 win rate	1.39	0.19	1.10	1.69
Personal 4231 use * personal 4231 win rate	-1.02	0.30	-1.50	-0.55
Personal 4231 use * personal win rate	-0.31	0.48	-1.08	0.47
Population 4231 use	1.17	0.60	0.12	2.03
Population 4231 win rate	0.28	0.28	-0.16	0.72
Population 4231 use * population 4231 win rate	0.09	0.80	-1.18	1.36
Population 4231 use * personal win rate	-1.62	0.65	-2.65	-0.56
Varying effects:				
Manager	0.56	0.08	0.44	0.68
Manager * personal 4231 use	1.46	0.16	1.21	1.72
Manager * population 4231 win rate	2.69	0.47	1.95	3.44
Division	0.20	0.16	0.02	0.48
Division * personal 4231 use	1.16	0.73	0.38	2.28
Division * population 4231 win rate	1.03	0.68	0.13	2.22

Table S7: Tests of the differences between varying effects from the real data and varying effects from randomised data, to test hypothesis H3 with X=20 day time window. Values shown are real minus randomised standard deviations.

	mean	sd	5.5%	94.5%
Manager * personal 4231 use	0.87	0.16	0.61	1.13
Manager * population 4231 use	2.40	0.39	1.76	3.00
Division * personal 4231 use	0.87	0.46	0.34	1.71
Division * population 4231 use	0.33	0.56	-0.34	1.29

Table S8: Tests of the differences between varying effects from the real data and varying effects from randomised data, to test hypothesis H3 with X=40 day time window. Values shown are real minus randomised standard deviations.

	mean	sd	5.5%	94.5%
Manager * personal 4231 use	1.13	0.19	0.83	1.43
Manager * population 4231 use	2.00	0.48	1.22	2.75
Division * personal 4231 use	1.23	0.64	0.47	2.42
Division * population 4231 use	0.50	0.60	-0.24	1.59

2.2 Hypothesis H2: ratio of population to personal 4231 use

For $X = 20$, the mean population:personal use ratio was 0.92, 89%CI[0.40, 1.63]. For $X = 40$ it was 0.68, 89%CI[0.16, 1.41]. For $X = 60$ it was 0.54, 89%CI[0.05, 1.25]. For all time windows, therefore, the ratio was not only not greater than one as was predicted, but the CIs are in all cases so wide that they encompass one. There is, however, a trend towards lower ratios with longer time windows, indicating that assuming longer time windows leads to greater reliance on personal information over population information.

2.3 Hypothesis H3: variation across managers and divisions

Tables S7-S9 show that for $X = 20$, $X = 40$ and $X = 60$, as for $X = 30$, there is more varation in 4231 personal and population use across managers than randomised data, and more variation across divisions in personal 4231 use, but not across divisions in population 4231 use.

Table S9: Tests of the differences between varying effects from the real data and varying effects from randomised data, to test hypothesis H3 with X=60 day time window. Values shown are real minus randomised standard deviations.

	mean	sd	5.5%	94.5%
Manager * personal 4231 use	1.27	0.20	0.96	1.59
Manager * population 4231 use	2.47	0.50	1.69	3.26
Division * personal 4231 use	1.07	0.74	0.27	2.20
Division * population 4231 use	0.81	0.72	-0.15	2.04

Table S10: Model estimates for the quadratic regression model with manager as unit of analysis, to test hypothesis H4 with X=20 day time window. Parameter a is the intercept, b1 is the linear coefficient and b2 the quadratic coefficient. Win rate is modelled as normally distributed with standard deviation sigma. See SI for priors.

	mean	sd	5.5%	94.5%
a	0.09	0.12	-0.10	0.30
b1	-0.11	0.14	-0.35	0.11
b2	0.02	0.04	-0.05	0.09
sigma	1.00	0.04	0.94	1.07

2.4 Hypothesis H4: population to personal use ratio and win rate

Tables S10-S12 show, as for $X = 30$, that there is no quadratic or linear relationship between manager win rate and population:personal use ratio for $X = 20$, $X = 40$ or $X = 60$.

Table S11: Model estimates for the quadratic regression model with manager as unit of analysis, to test hypothesis H4 with X=40 day time window. Parameter a is the intercept, b1 is the linear coefficient and b2 the quadratic coefficient. Win rate is modelled as normally distributed with standard deviation sigma. See SI for priors.

	mean	sd	5.5%	94.5%
a	0.04	0.14	-0.18	0.26
b1	-0.17	0.17	-0.44	0.10
b2	0.10	0.06	0.01	0.19
sigma	1.00	0.04	0.94	1.06

Table S12: Model estimates for the quadratic regression model with manager as unit of analysis, to test hypothesis H4 with X=60 day time window. Parameter a is the intercept, b1 is the linear coefficient and b2 the quadratic coefficient. Win rate is modelled as normally distributed with standard deviation sigma. See SI for priors.

	mean	sd	5.5%	94.5%
a	0.02	0.13	-0.19	0.23
b1	-0.11	0.14	-0.32	0.12
b2	0.06	0.03	0.01	0.10
sigma	0.99	0.04	0.94	1.06

3 Revised models excluding own team formations from population predictors

The following results are from the revised models which, for the population predictors, exclude formations used by the same team during the X-day window. That is, for team i, the population predictors are calculated from all formations used in the X-day window except those used by team i. This ensures a stricter separation between personal and population predictors, as now team i's formations are only used in the personal predictors, not the population predictors. This change had negligible effects on the results, and all conclusions for all hypotheses remain qualitatively identical to the original findings derived from the original preregistered analyses presented in the main paper.

3.1 Hypothesis H1: combination of population and personal information use

Table S13 shows almost identical model comparison for the revised models excluding own team from population predictors, compared to the original model given in the main text. Table S14 also shows very similar estimates for the full revised model compared to the original.

3.2 Hypothesis H2: ratio of population to personal 4231 use

For the revised model excluding own team from population predictors, the mean population:personal use ratio was 0.79, 89%CI[0.21, 1.48]. Similar to the original model, the mean ratio is less than one, and the CIs are so wide that they encompass one.

Table S13: Model comparison to test hypothesis H1, with revised models excluding own team from the population predictors.

	WAIC	pWAIC	dWAIC	weight	SE	dSE
Full model	12263.40	338.69	0.00	1	144.10	
Personal model	12336.32	297.62	72.92	0	144.13	15.21
Population model	13691.85	356.98	1428.45	0	136.00	92.38
Null model	14326.32	251.56	2062.92	0	134.84	101.64

Table S14: Parameter estimates for the full revised model excluding own team from the population predictors. Home/away is an indicator trait with separate estimates for formations used home and away. Varying effects show the standard deviations of the varying intercepts and slopes. See SI for full model specification and priors.

	mean	sd	5.5%	94.5%
Fixed effects:				
Home/away: Home	-0.02	0.16	-0.27	0.21
Home/away: Away	-0.14	0.16	-0.40	0.09
Team strength	0.03	0.27	-0.42	0.46
Personal 4231 use	2.12	0.67	0.90	2.99
Personal 4231 win rate	0.84	0.13	0.63	1.05
Personal 4231 use * personal 4231 win rate	-0.64	0.21	-0.97	-0.32
Personal 4231 use * personal win rate	0.04	0.34	-0.51	0.58
Population 4231 use	1.27	0.46	0.49	1.94
Population 4231 win rate	-0.15	0.20	-0.47	0.17
Population 4231 use * population 4231 win rate	-0.04	0.63	-1.05	0.98
Population 4231 use * personal win rate	-0.96	0.48	-1.73	-0.20
Varying effects:				
Manager	0.72	0.07	0.61	0.84
Manager * personal 4231 use	1.24	0.14	1.01	1.48
Manager * population 4231 win rate	1.95	0.44	1.27	2.61
Division	0.26	0.19	0.05	0.59
Division * personal 4231 use	1.32	0.66	0.58	2.54
Division * population 4231 win rate	0.64	0.49	0.05	1.54

Table S15: Tests of the differences between varying effects from the real data and varying effects from randomised data, to test hypothesis H3 with the revised model excluding own team from the population predictors. Values shown are real minus randomised standard deviations.

	mean	sd	5.5%	94.5%
Manager * personal 4231 use	1.13	0.16	0.86	1.39
Manager * population 4231 use	1.58	0.52	0.74	2.35
Division * personal 4231 use	1.23	0.67	0.48	2.48
Division * population 4231 use	0.41	0.55	-0.32	1.37

Table S16: Model estimates for the quadratic regression model with manager as unit of analysis, to test hypothesis H4 with the revised model excluding own team from the population predictors. Parameter a is the intercept, b1 is the linear coefficient and b2 the quadratic coefficient. Win rate is modelled as normally distributed with standard deviation sigma. See SI for priors.

	mean	sd	5.5%	94.5%
a	0.03	0.14	-0.19	0.26
b1	-0.15	0.18	-0.44	0.14
b2	0.09	0.07	-0.02	0.20
sigma	1.00	0.04	0.94	1.06

3.3 Hypothesis H3: variation across managers and divisions

Table S15 shows that for the revised model excluding own team from population predictors, there is more varation in 4231 personal and population use across managers than randomised data, and more variation across divisions in personal 4231 use, but not across divisions in population 4231 use. This is the same as the original model.

3.4 Hypothesis H4: population to personal use ratio and win rate

Table S16 shows that there is no quadratic or linear relationship between manager win rate and population:personal use ratio for the revised model, just as for the original model.

Table S17: Model comparison to test hypothesis H1, with revised models including additional opponent controls.

	WAIC	pWAIC	dWAIC	weight	SE	dSE
Full model	12242.47	339.23	0.00	1	144.06	
Personal model	12312.90	299.61	70.43	0	144.10	14.60
Population model	13525.10	359.51	1282.63	0	137.34	88.33
Null model	14291.80	252.77	2049.33	0	135.20	101.61

4 Revised models including additional opponent controls

The following results are for models that are identical to the preregistered original analyses presented in the main paper, but with two additional controls added to all models: (i) the relative strength of the opponent, calculated in the same way as the own team strength predictor, on the assumption that managers may alter their formations depending on opponent strength (e.g. play defensive formations against strong teams and attacking formations against weak teams); and (ii) the formation played by the opponent, coded as 4231 or non-4231, on the assumption that managers may attempt to counter 4231 with either the same formation, matching players in the same positions, or with a different formation in an attempt to break the 4231 domination. Including these controls had negligible effect on the parameter estimates, and did not qualitatively change conclusions regarding any of the hypotheses.

4.1 Hypothesis H1: combination of population and personal information use

Table S17 shows almost identical model comparison for the revised models including additional opponent controls, compared to the original model given in the main text. Table S18 also shows very similar estimates for the full revised model compared to the original. The two additional control variables both reliably predict a manager's use of 4231: managers are more likely to use 4231 against stronger opponents, and when opponents use 4231 themselves.

Table S18: Parameter estimates for the full revised model including additional opponent controls. Home/away is an indicator trait with separate estimates for formations used home and away. Varying effects show the standard deviations of the varying intercepts and slopes. See SI for full model specification and priors.

	mean	sd	5.5%	94.5%
Fixed effects:				
Home/away: Home	-0.07	0.16	-0.32	0.17
Home/away: Away	-0.21	0.16	-0.46	0.05
Team strength	0.00	0.27	-0.43	0.42
Opponent strength	0.79	0.16	0.55	1.04
Opponent 4231	0.14	0.05	0.06	0.21
Personal 4231 use	2.09	0.63	0.97	2.94
Personal 4231 win rate	0.84	0.13	0.63	1.06
Personal 4231 use * personal 4231 win rate	-0.62	0.21	-0.95	-0.28
Personal 4231 use * personal win rate	0.11	0.35	-0.45	0.66
Population 4231 use	1.27	0.47	0.45	1.94
Population 4231 win rate	-0.11	0.20	-0.43	0.22
Population 4231 use * population 4231 win rate	-0.59	0.66	-1.64	0.47
Population 4231 use * personal win rate	-1.12	0.51	-1.94	-0.31
Varying effects:				
Manager	0.72	0.08	0.60	0.85
Manager * personal 4231 use	1.25	0.14	1.02	1.48
Manager * population 4231 win rate	1.99	0.50	1.22	2.70
Division	0.27	0.19	0.05	0.59
Division * personal 4231 use	1.25	0.61	0.55	2.38
Division * population 4231 win rate	0.66	0.52	0.06	1.63

Table S19: Tests of the differences between varying effects from the real data and varying effects from randomised data, to test hypothesis H3 with the revised model including additional opponent controls. Values shown are real minus randomised standard deviations.

	mean	sd	5.5%	94.5%
Manager * personal 4231 use	1.14	0.16	0.88	1.41
Manager * population 4231 use	1.63	0.57	0.69	2.45
Division * personal 4231 use	1.17	0.62	0.45	2.31
Division * population 4231 use	0.43	0.57	-0.28	1.43

4.2 Hypothesis H2: ratio of population to personal 4231 use

For the revised model, the mean population:personal use ratio was -0.53, 89%CI[0.20, 1.32]. The mean is negative but this is unreliable and due to an extreme negative value. The confidence intervals, however, are similar to the original model, and widely encompass one indicating no support for the hypothesis.

4.3 Hypothesis H3: variation across managers and divisions

Table S19 shows that for the revised model with additional opponent controls, there is more varation in 4231 personal and population use across managers than randomised data, and more variation across divisions in personal 4231 use, but not across divisions in population 4231 use. This is the same as the original model.

4.4 Hypothesis H4: population to personal use ratio and win rate

Table S20 shows that there is no quadratic or linear relationship between manager win rate and population:personal use ratio for the revised model including additional opponent controls, just as for the original model.

Table S20: Model estimates for the quadratic regression model with manager as unit of analysis, to test hypothesis H4 with the revised model including additional opponent controls. Parameter a is the intercept, b1 is the linear coefficient and b2 the quadratic coefficient. Win rate is modelled as normally distributed with standard deviation sigma. See SI for priors.

	mean	sd	5.5%	94.5%
a	0.03	0.14	-0.19	0.25
b1	-0.17	0.17	-0.45	0.10
b2	0.11	0.07	0.00	0.21
sigma	1.00	0.04	0.94	1.06

References

1. McElreath, R. Rethinking: Statistical Rethinking Book Package version 1.88. (2019).
